# Prediction of the Young’s Modulus of Polylactic Acid Specimens Manufactured by Fused Deposition Modeling Using Machine Learning-Based Stacking Ensemble Methods

**DOI:** 10.3390/polym18131661

**Published:** 2026-07-04

**Authors:** Alexandru Constantin Stanciu, Anton Hadăr, Nicolae Goga, Mihai-Constantin Butolo, Florin Baciu, Stefan-Dan Pastrama, Daniel Vlăsceanu

**Affiliations:** 1 Department of Strength of Materials, Faculty of Industrial Engineering and Robotics, National University of Science and Technology POLITEHNICA Bucharest, Splaiul Independentei 313, 060042 Bucharest, Romania; stanciualexandru04@yahoo.com (A.C.S.); mihai.butolo@gmail.com (M.-C.B.); stefan.pastrama@upb.ro (S.-D.P.); daniel.vlasceanu@upb.ro (D.V.); 2 Technical Science Academy of Romania, Dacia Blvd. 26, 030167 Bucharest, Romania; 3 Academy of Romanian Scientists, Ilfov Street 3, 050045 Bucharest, Romania; 4 Department of Engineering in Foreign Languages, Faculty of Engineering in Foreign Languages, National University of Science and Technology POLITEHNICA Bucharest, Splaiul Independentei 313, 060042 Bucharest, Romania; nicu.goga@upb.ro

**Keywords:** additive manufacturing, Fused Deposition Modeling, polylactic acid, Young’s modulus, machine learning, stacking ensemble

## Abstract

In this paper, a machine learning model to predict the Young’s modulus of polylactic acid specimens manufactured by Fused Deposition Modeling is proposed, based on a stacked ensemble architecture. The model uses as input parameters the fill degree, printing speed, filling pattern, yield strength, and tensile strength, along with additional features obtained through feature engineering. The proposed approach integrates nine base models with a linear meta-model, allowing it to capture both linear and nonlinear relationships between the variables. The results obtained on the test dataset show strong predictive performance, with a Mean Squared Error with a value of 7.31 together with a Coefficient of Determination R^2^ with a value of 0.99, which is noticeably better than the performance of the individual models. To validate the model, a separate group of specimens was tested, and the difference between the measured and predicted Young’s modulus was about 1% on average. The model was also implemented in a desktop application with a graphical interface, in which the calculation can be run directly, thus allowing a rapid estimation of Young’s modulus. In this way, the need for laborious experimental testing is reduced with the help of AI-based approaches in additive manufacturing.

## 1. Introduction

### 1.1. Additive Manufacturing and the Mechanical Behaviour of 3D-Printed Parts

The growing need to produce complex, customizable, and high-performance products in an efficient way has promoted the use of three-dimensional printing or Additive Manufacturing (AM), which is an additive technique that uses successive layer deposition for producing three-dimensional objects. Among AM techniques, Fused Deposition Modeling (FDM) is currently the most common method thanks to the availability of affordable machines and the large number of available materials such as polymers, composites, and metallic alloys. The rapid growth of this technique is driven by its potential applications in developing durable engineering components, customized prosthetic implants, and advanced biomedical equipment [1]. For this reason, an increasing interest exists in studying the relationship among structure–process–properties of parts produced by FDM since these parts generally show poor mechanical performance when compared to those produced by traditional manufacturing techniques [2].

The use of AM has experienced increased usage throughout many different types of industries over the last few decades. AM has also been developed as a method for producing parts that have unique shapes with a higher degree of customization than traditional methods and less wasted materials; thus, it has evolved from a tool used primarily for creating prototypes into a general-purpose manufacturing process that can be applied to a broad array of materials [3]. From a market perspective, industrial AM is expected to expand considerably in the coming years. According to ADDere, its value could rise from USD 3.83 billion in 2023 to USD 16.93 billion by the year 2030, corresponding to an estimated compound annual growth rate of 23.6% [4].

Parts manufactured by 3D printing must meet requirements related to their elastic constants and mechanical properties (Young’s modulus, yield strength, tensile strength, etc.). These characteristics are influenced not only by the chosen material but also by the printing parameters. Thus, a wide range of properties can be obtained for 3D printable structures, but their experimental evaluation becomes difficult due to the countless combinations of parameters [5]. Research has also shown that the nozzle temperature and the cooling rate, among other parameters, can influence the mechanical properties of 3D-printed parts [6]. In recent years, the use of Machine Learning (ML) models in simulations has become a promising solution to this problem.

Predicting mechanical and elastic properties of the manufactured parts plays an increasingly important role in process optimization and in reducing the costs associated with experimental testing. Bayraktar et al. [7] showed that artificial neural networks (ANN) can accurately predict the tensile strength of Polylactic Acid (PLA) samples printed by FDM technology, achieving a coefficient of determination with an approximate value of 0.999 on controlled experimental sets. Similarly, Rooney et al. [8] used Bayesian Neural Networks to estimate the Young’s modulus and flexural strength of parts manufactured by Digital Light Processing technology, reporting errors below 5%. Advanced transfer learning approaches in additive manufacturing have shown promising results, with methods based on deep convolutional neural networks achieving 98.7% accuracy in predicting tensile strength and 93.1% in predicting elongation at break, thus validating the potential of ML techniques for real-time control of manufacturing processes [9]. Fetecau et al. [10] also used multi-output ANN models to study polyamide 12, as well as composites reinforced with carbon and glass fibers, predicting the Young’s modulus and the tensile strength with errors below 5%, on samples fabricated by FDM, and taking into account the printing orientation and the degree of filling.

### 1.2. Prediction of Mechanical Properties in FDM Additive Manufacturing

ML is concerned with methods that identify patterns in existing data and uses these patterns to generate predictions or decisions without human intervention for every specific situation. In engineering, ML methods are frequently used for classification (e.g., detecting defects on the production line), regression (e.g., predicting numerical values such as Young’s modulus), and clustering (e.g., identifying patterns or groupings in data without predefined labels).

Data-Driven ML in AM is very well represented, as it provides an effective method for process optimization, model development of large systems, and quality control from the preprocessing (AM design, parameters) through the processing (defects) phases, and finally in the post-processing (part quality) phase [11,12,13]. Defect detection and classification offer benefits such as monitoring print quality and preventing waste and excessive material costs, but some challenges include efficient sensor integration and real-time processing of complex data [14].

For predicting mechanical properties, the general approach is supervised learning in the form of a regression problem. Here, the model is given a set of input data and associated target values, like Young’s modulus in this research. The final goal is that, after training the model, it can predict those values for new, previously unseen sets of inputs.

The effectiveness of using ML algorithms to predict the mechanical properties of sheet metal in stamping processes was recently presented by Lee et al. [15], who compared five AI algorithms to predict the yield strength and uniform elongation (elongation where the maximum stress occurs in the stress–strain curve), achieving outstanding performances with two ML algorithms based on decision trees, namely Random Forest (R^2^ = 0.9921) and Gradient Boosting (R^2^ = 0.9903), thus validating the superiority of ensemble approaches in the field of material properties. Zhao et al. [16] presented a high-generalizability machine learning framework, combining a micro-mechanical orientation-averaging homogenization procedure with a stacked ensemble configuration to predict the macroscopic elastic and mechanical behavior of short-fiber reinforced polymer composites, by integrating Extra Trees, XGBoost, and LightGBM as base learners under an optimized meta-regressor. A stacking ensemble machine learning architecture to evaluate cyclic deterioration components in structural steel W-section beams under seismic loads was proposed by Khoshkroodi et al. [17]. The hierarchical predictive framework aggregates AdaBoost, Random Forest, and XGBoost as low-tier base regressors, using a meta-learner layer to systematically eliminate prediction errors across diverse geometric parameters. An advanced stacking ensemble configuration to predict the stacking fault energy (SFE) in high-manganese steels was presented by Tiwari et al. [18]. The framework utilizes individual, diverse algorithms, including kernel-based systems and gradient-boosted trees as base models, feeding their outputs into a specialized meta-regressor layer to mitigate overfitting and manage data heterogeneity across various metallurgical families. Han et al. [19] proposed a hierarchical stacking ensemble framework to predict the ultimate tensile strength, yield strength, and elongation performance of additively manufactured Ti6Al4V titanium alloys fabricated via large-scale laser powder bed fusion. By stacking heterogeneous machine learning structures, including artificial neural networks, gradient boosting, and kernel ridge regressions, the model accurately integrated printing process inputs, localized thermal history variations, and subsequent post-heat treatment boundaries. Adriyan et al. [20] proposed an optimized stacked generalization model to predict and simultaneously optimize the conflicting mechanical relationships between high tensile strength, yield strength, and elongation ductility across variable aluminum alloy series. They used instances of Extra Trees and CatBoost tuned via the Optuna hyperparameter framework and integrated by a linear meta-learner to obtain a model that successfully generalized performance traits across diverse chemical composition and temper datasets.

In engineering, the integration of ML has brought remarkable advantages since evaluation of the mechanical properties of materials manufactured by additive technologies by traditional methods requires the combination of a large number of factors, resulting in low efficiency and high costs. In this context, the use of ML strategies has gained increasing attention for the efficient prediction of these complex properties [21].

### 1.3. ML Approaches in Additive Manufacturing

Engineers constantly need to estimate how created parts will behave. This is where predictive models come in. Once trained with quality data, they become valuable tools in the decision-making process. The practical applicability of ML algorithms in predicting mechanical properties was found even in the construction industry, with recent studies showing that algorithms including Support Vector Machine, Random Forest, and Gradient Boosting can successfully predict complex properties of concrete, including Young’s modulus, compression strength, and thermal expansion coefficient [22].

A traditional approach to adjusting print properties typically requires creating and testing a new prototype for each adjustment. Adjusting and comparing multiple infill percentages, patterns, or printing speeds becomes expensive and time-consuming. The predictive model reduces the amount of labor required to compare these properties since it estimates the Young’s modulus based on the user-selected inputs. During the prototyping phase, there is often a necessity to adjust the design and/or the printing settings frequently. Utilizing the model allows users to evaluate changes to their printing configuration prior to making any additional specimens.

Each type of ML algorithm used in this area has its own distinct features. Support vector machines are good at processing small datasets and support variable amounts of input variables. Random Forest produces stable outputs most of the time; it supports identifying the relative importance of input variables and does so without requiring excessive computing resources. Neural networks, although capable of modeling complex nonlinear relationships, require significantly more data than the two previously mentioned algorithms in order to produce output that is considered reliable [23].

While the above-mentioned research in this field shows the strength of AI in additive manufacturing, it relies on simpler input parameters such as raster angle, as opposed to complex geometries such as hexagonal infill mode. Also, it requires more training data or uses additional hardware to generate images that are required for image classification models. Also, it should be noted that the specific combination of FDM-manufactured PLA specimens, Young’s modulus prediction, limited experimental data, Generative Adversarial Networks-based synthetic data augmentation, stacked ensemble modeling and independent experimental validation remains less extensively explored. That is why a reliable model, which can predict mechanical behavior just from the print settings and a couple of physical characteristics, becomes extremely useful. In this context, the present study develops and validates a stacking-based ML approach for estimating the Young’s modulus of PLA specimens manufactured by FDM technology. The advantage of using the proposed stacked methodology is that it takes the predictions from nine base models and combines their predictions into a meta model. This allows the meta model to benefit from the strengths of each of the linear, tree-based or ensemble models used while mitigating the weaknesses of each base model. Also, using GANs to generate synthetic data with the same statistical characteristics as the original dataset allows the models to have access to more training data, thus improving accuracy to some extent. Since the original dataset contained only 40 specimens that would need to be split into training and test data, there was a high risk that some parameter combinations could be completely excluded from the training data.

## 2. Materials and Methods

### 2.1. The Specimens

**Figure 1:**
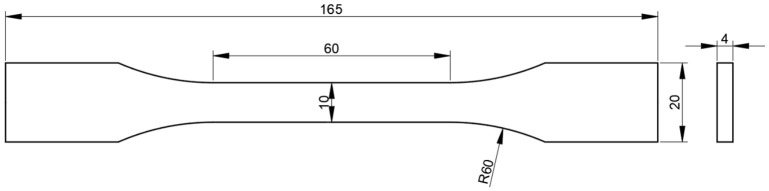
The ISO 527-2 dog-bone-type specimen.

Dog-bone-type specimens used for tensile testing of PLA were manufactured according to the standard ISO 527-2 [24], as shown in Figure 1. PLA was chosen for its high processability and high potential for medical applications thanks to the biodegradability and biocompatibility of this material [25,26]. PLA’s high processability comes from its low melting temperature, which allows good adhesion between layers [25]. PolyMax™ PLA filament, an easy-to-print type of filament with improved mechanical properties [27], was used to print the specimens. The experimental set comprised 40 specimens, organized into pairs of two for each combination of three input parameters: printing speed, infill pattern, and infill degree, thus allowing the repeatability of the process to be evaluated. A full factorial design was considered in the experiments. Every single possible combination was tested, and the average value of the obtained properties was considered. The results showed a very good repeatability for each pair of identical specimens, proving that the experimental procedure is accurate.

Each specimen received a code based on its printing settings: [Degree][Mode][Speed]_[Replica]. The first part gives the infill percentage, the letter shows the infill pattern (G for diagonal and F for hexagonal), and the speed is given in mm/s. For example, 80F80_2 means 80% infill, hexagonal pattern, and 80 mm/s printing speed. The value after the underscore shows the replicate number; here it is the second specimen for that setting.

### 2.2. The Process Parameters

Three printing parameters were studied in this paper: the infill level, the infill pattern, and the printing speed.

Three infill levels were considered in this research, namely 60%, 80%, and 100%. This parameter affects the weight of the specimen, material consumption, printing time, and mechanical response. Previous research reports that the Young’s modulus and tensile strength generally increase with infill density, especially at low and medium infill values [28]. At 100% infill, the printed structure is solid, which leaves no internal voids and limits possible stress concentration points. This means that at 100% fill level, the differences between the diagonal and hexagonal fill modes become irrelevant because the samples have the same internal structure. For this consideration and to save time and materials costs, only samples with 100% fill degree for the diagonal fill mode were printed.

The infill pattern defines the internal geometry of the part and influences how stresses are distributed, as well as its stiffness and material usage. Two representative configurations were employed for the specimens: diagonal and hexagonal. The diagonal infill pattern consists of rectilinear filaments deposited at approximately +45° and −45° with respect to the longitudinal axis of the specimen, resulting in a 90° angle between the raster directions of successive layers. This generates an orthogonal structure, which is relatively simple and fast to print and offers good strength in the directions of the grid lines, being one of the most used configurations in practice. The hexagonal fill mode is a honeycomb structure, inspired by nature, and recognized for its great structural efficiency and good ratio between strength and material consumption. The hexagonal geometry distributes stresses evenly in all directions of the plane, offering nearly isotropic properties in the plane of the specimen. For the configuration with 100% fill, only the diagonal mode was investigated since in this case the internal structure becomes practically solid, and the geometrical differences between different fill modes become less relevant. As emphasized in the technical guide of Prusa Research [29], solid models do not generally offer mechanical properties significantly superior compared to less dense fill modes, but they consume more filament and require more printing time. Moreover, at high densities, the rectilinear model becomes the standard recommended by major additive manufacturing software.

The print speed directly influences the fabrication time, the quality of the inter-layer bonds and the final mechanical properties. Four levels of speed were investigated, namely 40 mm/s, 60 mm/s, 80 mm/s and 100 mm/s. Lower speeds favor a better thermal fusion between the deposited filaments, allowing superior molecular diffusion at the interface between layers and improved mechanical cohesion. Meanwhile, higher speeds allow a shorter production time, but can compromise the quality of the inter-layer adherence caused by insufficient time for complete fusion of the material. Studies showed a reduction in the Young’s modulus and other properties of the PLA specimens as a result of the increase in the printing speed. This was found to be a consequence of the lack of time to achieve an adequate bond between layers, as well as formation of porosities and micro-cracks [30].

### 2.3. The Testing Methodology

**Figure 2:**
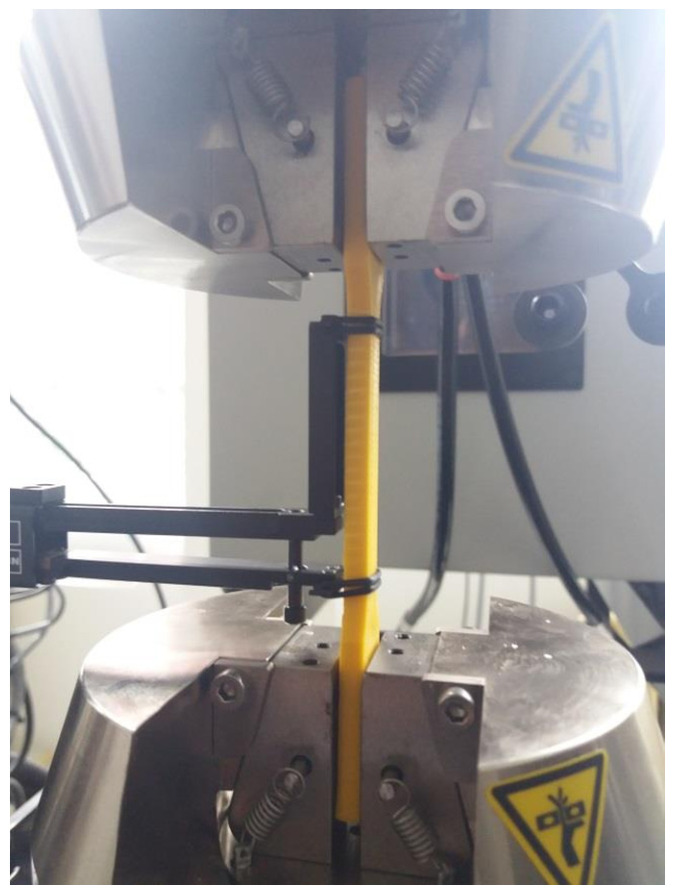
The tensile test.

**Figure 3:**
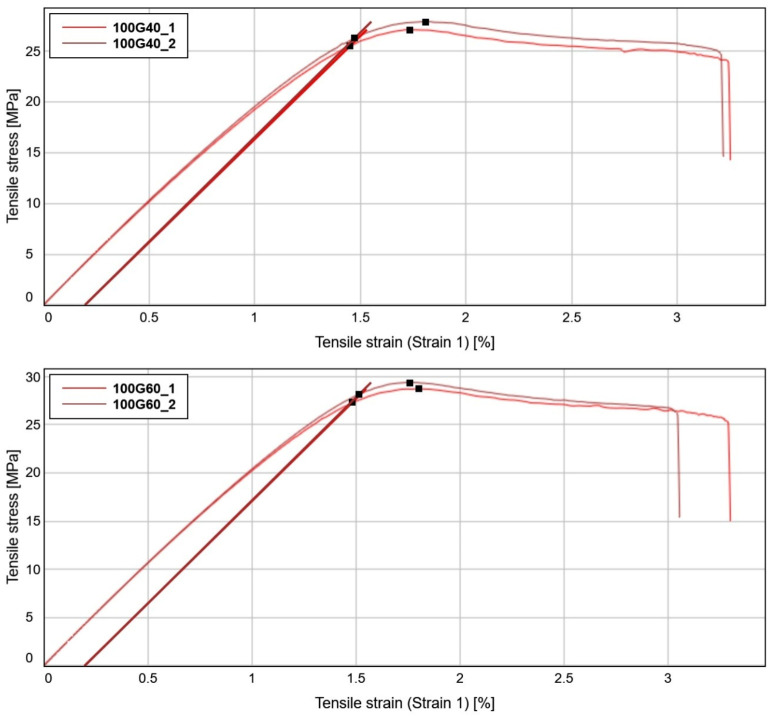
Stress–strain curves of samples with diagonal filling mode for different parameters (100% filling degree, printing speed 40/60 mm/s).

All tensile tests were carried out on an INSTRON 8872 universal testing machine (Instron, Norwood, MA, USA), equipped with hydraulic jaws and an extensometer mounted on the specimen to record the displacement Δ*L*, as shown in Figure 2. Static tests were performed with a monotonically increasing load until failure, and the tensile forces and corresponding displacements were automatically recorded to obtain the stress–strain curve. Some of the obtained stress–strain curves are shown in Figure 3.

### 2.4. Results of the Tensile Tests

For each specimen, the Young’s modulus, yield strength, and ultimate tensile strength were determined from the stress–strain curves. Representative samples of the input parameters and the obtained results are listed in Table 1. It can be noticed that the Young’s modulus *E* varied between approximately 1180 MPa and 2130 MPa, depending on the printing configuration; the yield strength σ*_y_* had values between 14.6 MPa and 28.1 MPa, while the values of the tensile strength σ*_u_* were between 15.7 MPa and 29.4 MPa. These values confirm previous studies, which have shown that these properties are strongly influenced by the parameters of the FDM process, especially the infill pattern, the thickness of the layers and the print speed. Specimens with aligned filaments in the direction of the stress have shown superior performance compared to those printed vertically [31].

The experimental results confirmed that the infill degree has the biggest impact on all investigated properties. Reducing the fill degree from 100% to 60% led to drops of about 40% in the values of the Young’s modulus and the tensile strength. This direct correlation between the infill degree and the mechanical properties is in line with other results from the scientific literature, which report significant increases in the tensile strength and stiffness with the increase in the infill degree [32].

For each printing configuration, results for the second specimen printed with the same parameters showed good repeatability, with differences remaining below 5%, which indicates that both the printing process and the mechanical tests were consistent. This is important for model validation because the training data should reflect the real behavior of the material and not random testing errors.

### 2.5. Prediction Methodology

The main objective of this research is to predict the Young’s modulus by designing and training a model that can estimate this property from its manufacturing parameters and key mechanical properties. The input variables used in the model are infill degree, printing speed, infill pattern, yield strength, and tensile strength, while the target variable of the model is the value of the Young’s modulus.

To achieve this goal, the experimentally obtained data is first prepared through a preprocessing stage that includes encoding the categorical variables into numerical form and scaling the continuous variables in order to reduce the influence of extreme values. Turning the categorical variables into numerical form is required because the ML algorithms cannot process text values such as “diagonal”. Scaling is required to bring the continuous values of different properties to the same scale. Scaling prevents variables with larger values from having a bigger influence on the training process. The final phase of the preprocessing phase is to generate additional relevant characteristics that can improve the accuracy of the models.

Next, the dataset needs to be split into two subsets: the training set and the test set. The training set is used to train the models, allowing them to learn existing patterns present in the data, while the test set is used only to objectively evaluate the performance of the model on never-before-seen data.

In the final step, the data is used to train and refine the predictive models using the stacked approach, called “stacking ensemble”. This method involves combining the results of several individual models into a meta-model, which learns to integrate and weigh the initial predictions, leading to superior results in comparison with the use of isolated models.

### 2.6. Preprocessing and Feature Engineering

Preprocessing is a set of operations applied to the data before it is used to train the model. In the context of the proposed algorithm, several techniques were used to enhance the quality of the input dataset.

Categorical variables were processed using two encoding approaches. Label encoding was used for categorical values such as “diagonal” and “hexagonal,” turning them to numerical values so these values could be introduced into algorithms that work only with numbers. One-hot encoding was also applied, creating separate binary columns for each category. This was useful especially for the linear models, where the categories should not be treated as ordered values.

From the array of available scaling methods, RobustScaler [33] was selected instead of StandardScaler [33] or MinMaxScaler [33]. This choice was made because the dataset contained variables with very different numerical ranges, for example Young’s modulus values in the order of thousands of MPa and tensile strength values in the order of tens of MPa. RobustScaler transforms the variables using the median and the interquartile range, which makes it less sensitive to very high or very low values than methods based on the mean, standard deviation, minimum, or maximum. This helped to obtain a more stable numerical representation of the input data before model training.

Feature engineering was included in the preprocessing stage. Some new variables were derived from the original inputs, such as squared terms, in order to represent nonlinear effects. Interaction terms were also added when two variables were expected to influence the result together.

These preprocessing operations are recommended when working with ML algorithms as a means to sanitize the data. These operations have two roles in any algorithm that uses ML: first, they make the relationships between variables easier to analyze, and second, they also help improve the accuracy of the predictive models.

### 2.7. Synthetic Data Generation Using Generative Adversarial Networks

When dealing with a limited number of tested specimens, a second data augmentation phase was added prior to training the prediction model. Typically, obtaining large experimental datasets in engineering is very difficult because each additional data point requires some form of material usage, manufacturing cost, time for mechanical testing and/or other costs associated with creating a new test specimen.

By augmenting the synthetic data generation process via a GAN, which consists of two neural network elements: a generator and a discriminator, the limitations of the small size of the experimental dataset were further reduced. During the training of the GAN architecture, both the generator and discriminator were trained at the same time. The generator produced synthetic samples of data that were consistent with the statistical distribution learned from the experimental data, while the discriminator attempted to determine whether the received samples were from either the original real data or the artificially created data. By virtue of the competitive nature of these two components during training, the generator creates samples of synthetic data that mimic those found in the original experimental dataset.

Synthetic data were generated through this augmentation process to provide additional combinations of process parameters and mechanical properties that mimicked the relationships discovered in the actual specimens. Since all manufacturing parameters as well as all mechanical properties from the original dataset were used as inputs, this augmentation step did not attempt to replace experimental testing but rather to assist in training the predictive models with increased amounts of data. Many ML algorithms, including many ensemble-based techniques, require a larger and more diverse training set. However, since the generated values cannot exist outside of the physical and technical boundaries established by the real experiments, they could be used only in conjunction with the parameters examined during the course of investigation.

**Figure 4:**
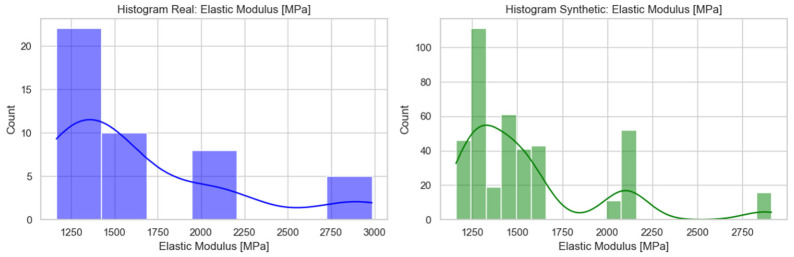
Histogram comparisons between real and synthetic values for Young’s modulus (the line on top of the chart is the kernel density estimate (KDE), used to visualize the smoothed distribution of the data).

As shown in the histogram from Figure 4, both datasets, the real and the synthetic one, exhibited comparable distribution shapes for Young’s modulus, demonstrating the GAN’s ability to understand and replicate the original dataset. As can be noticed in Figure 4, the Young’s modulus that results from the printing parameters has some gaps in the original dataset because only four printing speeds and three fill degrees were considered in the initial experiment. Other values could be obtained if more values of the printing parameters had been considered, but in this case, the material consumption and time needed to print additional samples would be much higher. The GAN generates new synthetic data designed to enrich existing data and minimize the chances that some of the original printing parameters are left out of the training process. But the GAN does not invent new values for the printing parameters; this is not its role, so the synthetic samples show the same gaps in the diagram on the right. The predictive models learn to estimate unseen data, but their accuracy inside missing ranges does improve when more data from those ranges becomes available.

The augmented dataset was then used in the next modeling stage, where several regression algorithms were trained individually and later combined through a stacked ensemble architecture. In this way, the GAN stage contributed to improving the data foundation of the prediction model, while the stacked ensemble was responsible for combining the strengths of the individual regressors and producing the final estimate of Young’s modulus.

### 2.8. Basic Models and Assemblies

After preprocessing and data augmentation, several regression algorithms were trained and compared as base models. The selected models were Ridge Regression, Polynomial Ridge Regression, Huber Regressor, Decision Tree Regressor, Random Forest Regressor, Extra Trees Regressor, Gradient Boosting Regressor, Bagging Regressor, and K-Nearest Neighbors Regressor [33]. These algorithms were chosen because they represent different modeling families, including linear models, tree-based models, ensemble methods, and distance-based methods. These basic models, each with their own strengths and limitations, are initially trained individually on the dataset. They are then integrated into the Stacking stage, an advanced technique that will be detailed in the next section. Stacking is more than a simple average of the model outputs. It uses a second-level model to combine the predictions of the individual algorithms. In this way, the ensemble can make use of the strengths of each model while reducing some of their individual weaknesses. The aim is to obtain a final model that is more accurate and stable than the separate models.

### 2.9. Stacked Ensemble: Principles and Implementation

Wolpert first introduced the stacking principle and has shown its effectiveness in reducing error rates [34]. Breiman [35] also demonstrated the benefits of the stacking principle and showed that stacking dissimilar predictors brings the greatest improvements in accuracy. The stacking ensemble principle has shown good results in predicting mechanical properties of alloys (HEAs) [36], surface roughness and tensile strength in PLA specimens [37], and dynamic compressive strength of brittle engineering materials [38].

To predict Young’s modulus, a stacked ensemble architecture was used. Several base models were combined through a “meta-model”, which learned how to take advantage of their individual predictions. This approach helped to reduce prediction variability and took advantage of the different strengths of the selected algorithms [29]. Unlike averaging or voting methods, stacking uses a separate model to decide how the outputs of the base models should be combined.

The stacked ensemble was built using cross-validation. First, the training data was divided into several folds of similar size. At each iteration, one fold was used for validation, and the others were used for training. This allowed all data points to be used in both training and validation, giving a more reliable evaluation of how the model performs on unseen data.

In the second stage, each base model was trained several times, each time leaving out one fold. The model then predicted the values for the fold that was not used during training. This out-of-fold procedure was repeated until every training instance had a prediction from each base model. These predictions were then organized into a matrix, with one row for each training instance and one column for each base model.

The ensemble included the nine base models presented in the previous paragraph. Their individual results, together with the result of the stacked ensemble, are reported in Table 2. The stacked ensemble was evaluated on the independent test set using the Mean Squared Error (MSE) and the Coefficient of Determination (R^2^) because it represents the final predictive model of the study. The individual base models were evaluated using cross-validation ± standard deviation, and their results are reported as CV MSE ± SD. This allows comparison not only of their average prediction error but also of their stability across different validation folds. The models were chosen to cover different types of behavior: linear models for easier interpretation, tree-based models for nonlinear effects and interactions, Huber Regressor for reduced sensitivity to outliers, and K-Nearest Neighbors Regressor for similarity-based prediction in the feature space.

Next, the output from the base models was combined with the true target values to serve as training data for the “meta-model”. In this study, linear regression was used as the meta-model. Its purpose was to learn the best way to use base-model predictions to produce a more accurate final estimate.

In the final stage, each base model first produced a prediction for the test samples or for any new input data. These predictions were then passed to the trained meta-model, which combined them into the final predicted value.

The main advantage of stacking is that the meta-model can adjust for the systematic errors made by the individual base models. This usually results in more stable predictions and better overall performance. The efficiency of the stacking technique is maximized when the base models are more diverse, bringing complementary perspectives on the input data and capturing different types of relations inside the dataset.

In conclusion, the proposed prediction methodology follows six essential steps. It starts by reading and preprocessing the data (scaling, encoding, and creating new variables). Further, data is split into a training set equal to 80% of the total data, used to train the model, and a test set representing 20% of the data, used to correctly evaluate the performance. Then, various base models are trained and combined using stacked ensemble techniques, using predictions on the excluded parts to train a meta-model, which integrates the individual results. The performance is then evaluated using evaluation metrics, such as MSE and R^2^. Finally, the components of the system are saved for further use.

## 3. Results and Discussions

**Figure 5:**
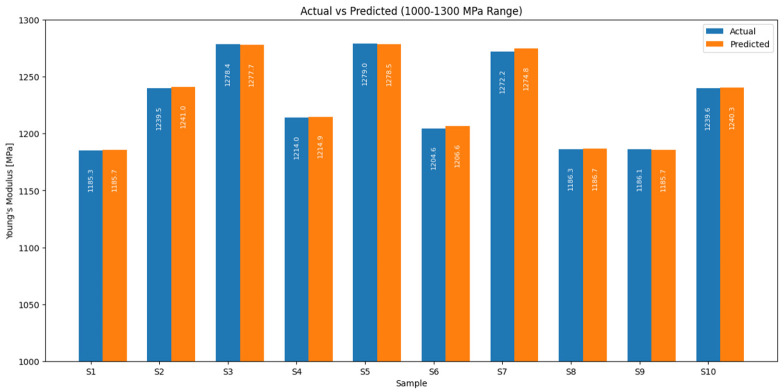
Correlation between actual values and values predicted by the stacked ensemble model.

To assess the performance of the model, two standard metrics traditionally used to evaluate regression models were employed: MSE and R^2^. Both are calculated using the data from the test set. MSE quantifies the average squared deviation between the measured values and the model predictions. Larger prediction errors contribute more strongly to the final MSE value. Moreover, for basic models, the scores resulting from cross-validation were also analyzed, which allowed for a more robust comparison between the algorithms. The correlation between the measured and predicted values is presented in Figure 5, where results for 10 random samples from the 1000–1300 MPa range of predicted Young’s modulus are depicted. It should be mentioned that the GAN generated 400 samples, out of which 80% were used for training and 20% for testing. This means that the test set consisted of 80 samples for which values for predicted versus actual values of Young’s modulus were obtained. It would be impossible to represent on a bar chart all 80 samples. Thus, only a subset of 10 samples from the test set are shown to highlight the very small difference between actual and predicted Young’s modulus.

The results suggest the advantage of the ensemble architecture. Looking at the results from Table 2, the final stacking model achieved an MSE equal to 7.31 and an R^2^ of about 0.9999, indicating a very high predictive capacity for the analyzed data. In comparison, the best-performing individual model was Extra Trees, with a CV MSE of approximately 15.51 ± 7.23, followed by Bagging, with 16.39 ± 4.78, and the simple polynomial model, with 19.19 ± 4.17. These results suggest that the stacking approach provided a lower prediction error than the individual models and that the meta-model was able to combine useful information from the base algorithms.

The Random Forest, Decision Tree and Gradient Boosting models provided intermediate performance, while Ridge Regression and Huber Regressor performed worse individually. However, their presence as a whole is necessary. On the contrary, it is precisely this diversity of perspectives that allows the meta-model to capitalize on both linear trends and local deviations, nonlinear behaviors, or robustness to extreme observations.

The feature relevance analysis showed that infill degree and tensile strength had the strongest influence on the prediction of Young’s modulus. In addition to the original infill degree, a squared term of this variable was introduced during feature engineering. This quadratic term allowed the model to represent nonlinear effects, meaning that the increase in stiffness was not assumed to be strictly proportional to the increase in infill degree. This is physically reasonable, because at high infill densities, the internal structure approaches a nearly solid configuration, and further increases in infill may produce smaller changes in Young’s modulus.

### 3.1. Experimental Validation of the Model

**Figure 6:**
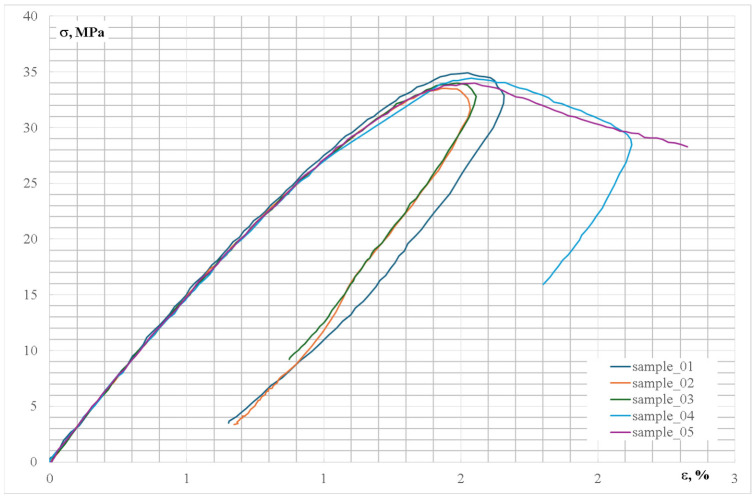
Specific stress–strain curves for the five specimens used in the validation process.

Beyond the performance obtained in the development phase, the practical relevance of the model was verified through an independent experimental validation. For this purpose, five additional specimens were manufactured, using the same process parameters and the same equipment as for the initial set. For the experimental validation, a representative configuration for the superior zone of the investigated domain was chosen, namely the 100% fill rate, diagonal fill mode, and print speed of 100 mm/s. After the fabrication process, the specimens were subjected to the same tensile tests on the same machine, and the experimental values of yield strength and tensile strength, together with the known print parameters, were introduced in the model to estimate Young’s modulus. The stress–strain curves for the additional specimens are presented in Figure 6, showing a good overlap in the elastic range and a reduced dispersion of the values of yield strength and tensile strength, which confirms the repeatability of the mechanical response for the analyzed configuration.

Very good accuracy was observed between the experimentally obtained and predicted values listed in Table 3.

For the five specimens, the measured Young’s modulus had an average value *E_exp_* = 2900.69 MPa, while the average predicted value was *E_p_* = 2893.81 MPa, with an average error of 1.03%, and the individual errors ranging between 0.21% and 2.49%. The largest deviation was associated with the sample that had the highest Young’s modulus, with a value of 2984.23 MPa, a value that is outside the dominant zone of the training data. Even so, the prediction still remained inside acceptable limits of engineering use.

### 3.2. Complementary Prediction Model Based Only on the Printing Parameters

Besides the model that was presented above, a complementary variant that uses only printing parameters as input variables was developed and analyzed. In this configuration, the model uses infill degree, fill mode and print speed, while yield strength and tensile strength are eliminated from the input dataset. The output variable remains Young’s modulus.

This variant is useful in the preliminary design stage, when only the printing conditions are known. The hybrid model presented before is applicable in cases where the yield strength and tensile strength are already available from previous tests, internal databases, technical data sheets or specialized literature for similar materials and printing conditions. Therefore, using yield strength and tensile strength does not necessarily require additional uniaxial tensile tests.

This model was trained on the synthetic dataset and then tested on the initial experimental dataset, which contains 40 specimens. The results are presented in Table 4.

While the errors are higher than in the hybrid model, the high value of the coefficient of determination (R^2^) shows that the printing parameters contain sufficient information to accurately estimate Young’s modulus. The two variants should be viewed as complementary, the hybrid model offering higher precision when more mechanical properties are available, and the model based on just the print settings can be used in the preliminary phase before uniaxial tests.

### 3.3. The Graphical Application

To make the proposed solution usable in practice, a desktop application with a graphical interface was developed in Python (https://www.python.org/, accessed on 24 May 2026). The aim was not only to demonstrate the functionality of the algorithm but also to turn it into a tool that can be used by engineers and operators without extensive experience in programming or data analysis.

The application was organized in a modular way, with three main components: a loading module that restores the trained models and the objects used for scaling and encoding, a prediction module that performs data processing and generates results, and a graphical interface that handles user interaction.

**Figure 7:**
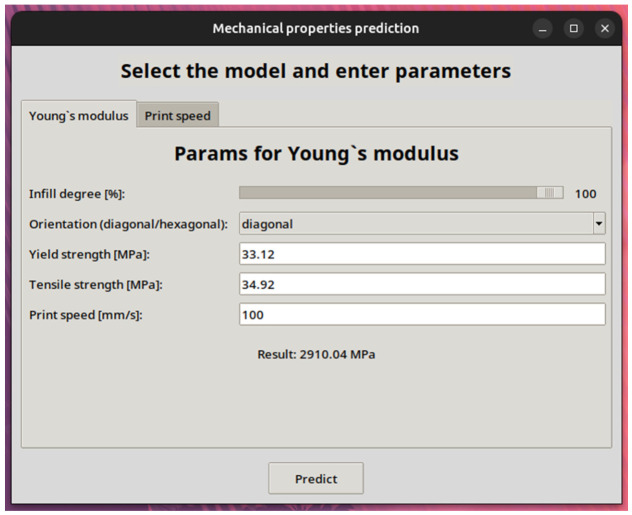
Graphical interface used for longitudinal modulus of elasticity prediction.

The graphical interface shown in Figure 7 is organized as a tabbed layout with two main panels: one dedicated to predicting the Young’s modulus and the other to predicting the optimum print speed. Both panels use the same type of input controls for the process parameters and mechanical properties. The infill degree is selected with a slider, the infill pattern can be chosen as either diagonal or hexagonal, and the yield strength and tensile strength are entered in separate numeric input fields.

## 4. Conclusions

This paper presents the development, evaluation, and validation of a model for predicting Young’s modulus for PLA specimens manufactured by FDM technology, using a stacked ensemble architecture. By combining nine basic models with a linear meta-model, the proposed approach was able to capture both the linear and nonlinear components of the relationship between the process parameters and the mechanical properties of the material. The numerical results revealed very high performance, with the stacked model achieving an MSE equal to 7.31, compared to the best-scoring base model, which had an MSE of 15.51. The stacked model also achieved an R^2^ value of 0.9999, with a clear advantage over the individual models.

The feature analysis showed that infill degree and tensile strength had the strongest influence on stiffness prediction. The derived features also helped describe the nonlinear behavior of the system more accurately. Experimental validation on five independent specimens showed that the model remained accurate after the development stage, with an average error of 1.03%, indicating very good potential for practical use.

A complementary variant of the model was also evaluated. This variant was based exclusively on the printing parameters and obtained R^2^ = 0.9969 on the real initial dataset. This shows that the methodology can be used as a hybrid high-precision model when yield strength and tensile strength are available, or as a preliminary estimation instrument based solely on printing conditions.

The proposed dataset was chosen to cover a wide array of printing parameters and to show the effectiveness of the prediction algorithms. For specific use cases, which may, in practice, require a higher or lower Young’s modulus, or smaller or higher printing parameters, the user can create an optimized dataset and retrain the algorithm to get updated results.

An important result is the transfer of the model from the theoretical algorithmic analysis to a practical tool, shaped as a desktop application with a graphical interface. With this implementation, the prediction method becomes available to users in the industrial environment and can contribute to the reduction of experimental tests, the optimization of process parameters and the standardization of technological decisions.

In addition to the predictive accuracy, the developed flow has value as a support tool for engineers because it connects the experimental process, data processing, and decision support all in one flow. This is particularly relevant in contexts where the rapid selection of parameters, the reduction of experimental tests and repeatable evaluation procedures are important. Thus, the study supports larger integrations of ML methods in additive manufacturing, not just as analytical models, but as operational tools that can help in the planning process and quality-oriented decisions.

As future development directions, the applicability of the proposed methodology can be extended to other materials and other additive manufacturing technologies, to integrate additional process parameters—like extrusion head temperature and layer thickness—as well as validating more complex geometries. Overall, the obtained results show that ensemble learning methods, sustained by experimental validation and integrated in an easy-to-use interface, represent a viable solution for estimating the mechanical properties in additive manufacturing.

## Figures and Tables

**Table 1:** Sample of the initial dataset.

Code	Infill Degree (%)	Infill Pattern	Printing Speed (mm/s)	Young’s Modulus (MPa)	Yield Strength (MPa)	Tensile Strength (MPa)
100G100_1	100	diagonal	100	2076.8	26.42	28.01
80G100_1	80	diagonal	100	1599.18	19.37	21.55
60G100_1	60	diagonal	100	1303.56	16.00	17.50
80F100_1	80	hexagonal	100	1408.43	17.63	18.57
60F100_1	60	hexagonal	100	1232.06	15.79	16.75

**Table 2:** Performance of the overall model and the main baseline models.

Model	Indicator	Value
Stacked Ensemble	MSE/R^2^	7.31/0.9999
Extra Trees	CV MSE ± SD	15.51 ± 7.23
Bagging	CV MSE ± SD	16.39 ± 4.78
Polynomial Ridge Regression	CV MSE ± SD	19.19 ± 4.17
KNN	CV MSE ± SD	76.62 ± 16.77
Random Forest	CV MSE ± SD	375.50 ± 33.35
Decision Tree	CV MSE ± SD	378.91 ± 35.52
Gradient Boosting	CV MSE ± SD	477.11 ± 51.54
Ridge Regression	CV MSE ± SD	1670.52 ± 232.86
Huber Regressor	CV MSE ± SD	7006.46 ± 2248.04

**Table 3:** Results for the additional specimens.

Sample	*E_exp_* [MPa]	σ*_u_* [MPa]	σ*_y_* [MPa]	*E_p_* [MPa]	Error [%]
1	2984.23	34.92	33.12	2910.04	2.49
2	2880.89	33.51	32.85	2886.88	0.21
3	2865.20	34.01	33.10	2901.05	1.25
4	2862.85	34.42	32.25	2879.20	0.57
5	2910.29	33.98	32.75	2891.89	0.63
*Average*	*2900.69*	*34.17*	*32.83*	*2893.81*	*1.03*

**Table 4:** Performance of the predictive model with the printing parameters as input.

Model	Input Parameters	MSE (MPa^2^)	RMSE (MPa)	MAE (MPa)	R^2^
Stacked ensemble with 3 parameters	Infill degree, fill mode, print speed	578.47	24.05	17.55	0.9969

## Data Availability

The original contributions presented in this study are included in the article. Further inquiries can be directed to the corresponding authors.
